# Belt and braces: Two escape ways to maintain the cassette reservoir of large chromosomal integrons

**DOI:** 10.1371/journal.pgen.1011231

**Published:** 2024-04-05

**Authors:** Egill Richard, Baptiste Darracq, Eloi Littner, Gael A. Millot, Valentin Conte, Thomas Cokelaer, Jan Engelstädter, Eduardo P. C. Rocha, Didier Mazel, Céline Loot

**Affiliations:** 1 Institut Pasteur, Université Paris Cité, CNRS UMR3525, Unité Plasticité du Génome Bactérien, Paris, France; 2 Sorbonne Université, ED515, Paris, France; 3 Institut Pasteur, Université Paris Cité, CNRS UMR3525, Microbial Evolutionary Genomics, Paris, France; 4 DGA CBRN Defence, Vert-le-Petit, France; 5 Institut Pasteur, Université Paris Cité, Bioinformatics and Biostatistics Hub, Paris, France; 6 Institut Pasteur, Université Paris Cité, Plateforme Technologique Biomics, Paris, France; 7 School of the Environment, The University of Queensland, Brisbane, Australia; Indiana University Bloomington, UNITED STATES

## Abstract

Integrons are adaptive devices that capture, stockpile, shuffle and express gene cassettes thereby sampling combinatorial phenotypic diversity. Some integrons called sedentary chromosomal integrons (SCIs) can be massive structures containing hundreds of cassettes. Since most of these cassettes are non-expressed, it is not clear how they remain stable over long evolutionary timescales. Recently, it was found that the experimental inversion of the SCI of *Vibrio cholerae* led to a dramatic increase of the cassette excision rate associated with a fitness defect. Here, we question the evolutionary sustainability of this apparently counter selected genetic context. Through experimental evolution, we find that the integrase is rapidly inactivated and that the inverted SCI can recover its original orientation by homologous recombination between two insertion sequences (ISs) present in the array. These two outcomes of SCI inversion restore the normal growth and prevent the loss of cassettes, enabling SCIs to retain their roles as reservoirs of functions. These results illustrate a nice interplay between gene orientation, genome rearrangement, bacterial fitness and demonstrate how integrons can benefit from their embedded ISs.

## Author summary

Bacteria have a great capacity to adapt to a wide range of stresses, such as the presence of antibiotics. The molecular mechanisms leading to this adaptation very often result in antimicrobial resistance phenomena responsible for the frequent failure of antibiotic treatments. Integrons are genetic systems that are largely involved in these adaptive responses to stress. They can carry up to 300 cassettes encoding different adaptive functions. It is therefore important to understand how such a large number of cassettes can be maintained during evolution. Here, we elucidate the question of integron maintenance by showing that bacteria can use two distinct escape ways to counter a too high cassette excision rate that could lead to an irreversible loss of functions. Firstly, by selecting mutated inactive integrases and secondly by selecting rearrangements within the integron that place the cassettes in a configuration where they become weakly excised. The presence of homopolymers in the integrase gene, as hotspots for replication sliding, favors the first escape solution, while the second is made possible by the presence of repeated insertion sequences integrated into the cassettes and allowing homologous recombination events. This work reveals the full power of the integron system maintaining its adaptive cassette reservoir.

## Introduction

**Fig 1:**
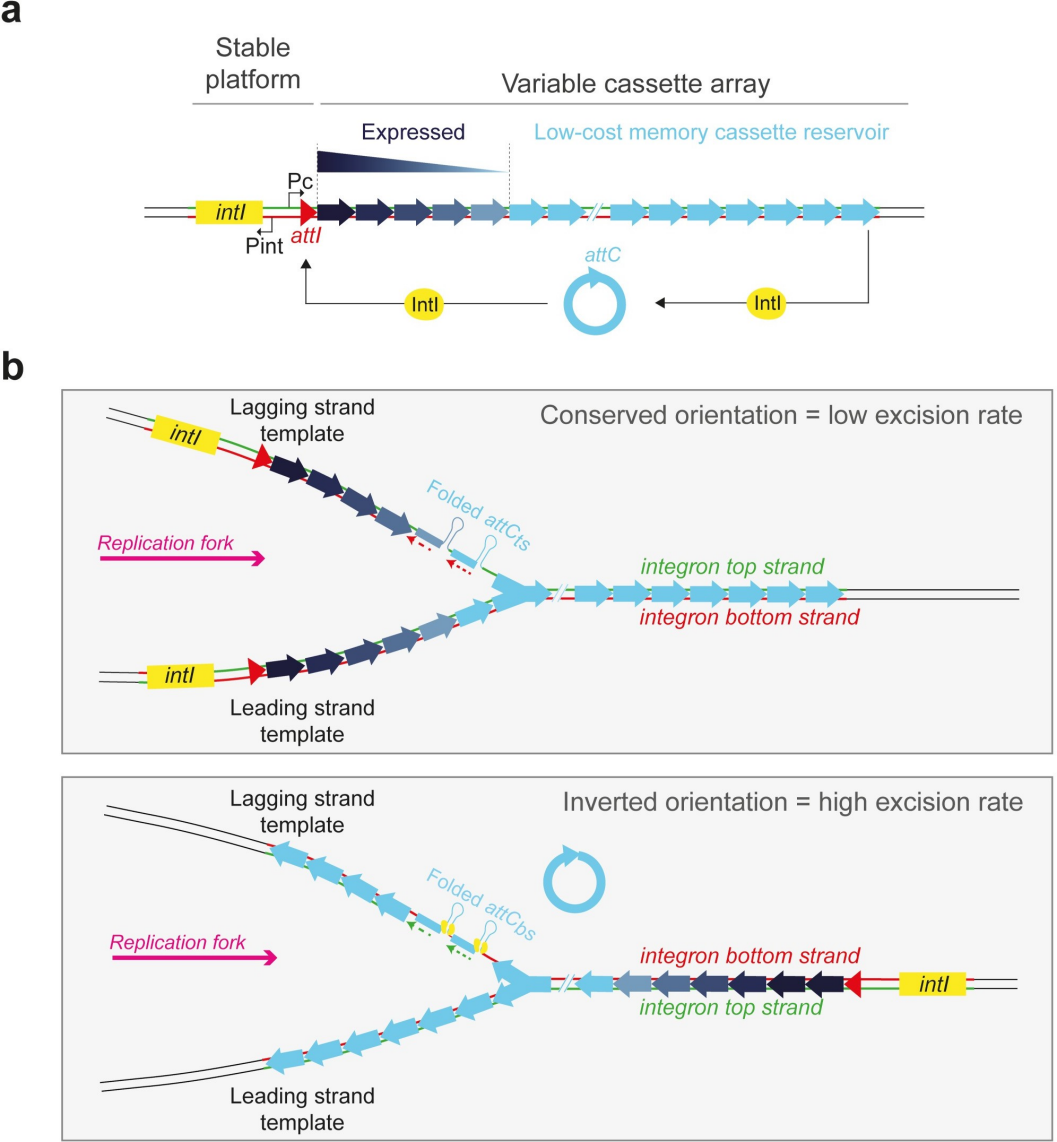
The integron system and the importance of its orientation towards replication. **a.** Schematic representation of the integron. The stable platform consists of a gene encoding the integrase (*intI*, in yellow) and its P_int_ promoter, the cassette insertion site *attI* (in red) and the cassette promoter P_C_ driving the expression of the downstream cassettes along an expression gradient. The cassettes are represented by arrows whose heads represent the *attC* site. Their color intensity represents their level of expression from dark blue (highly expressed) to light blue (not expressed). Cassettes can be excised through an *attC* × *attC* intramolecular reaction and be reintegrated in 1^st^ position of the integron array (near the P_C_ promoter) through an *attC* × *attI* intermolecular reaction. Then, cassettes become expressed. **b.** Mechanistic insight on the issue of integron orientation. Array of cassettes are represented while they are replicated. Their orientation towards the replication fork is indicated by the direction of the arrowhead representing the cassette. In their conserved orientation, SCI recombinogenic bottom strands of *attC* sites (*attC*_*bs*_) are carried by the continuously replicated leading strand template which supposedly limits their structuration. The non-recombinogenic top strands of *attC* sites (*attC*_*ts*_) are carried by the lagging strand template containing stretches of ssDNA (between the Okazaki fragments, dotted lines) which supposedly favors their structuration. In the inverted orientation, recombinogenic *attC*_*bs*_ are carried by the lagging strand template. A more frequent structuration of these *attC* strands is expected to lead to increased binding of the integrase and higher cassette excision rate.

Integrons are ancient genetic structures that play a crucial role in the spread of multidrug resistance in gram-negative bacteria and more generally in bacterial evolution [1–4]. These bacterial recombination systems can capture, store and shuffle small mobile elements–cassettes–encoding adaptive functions. They are organized in two parts: a stable platform and a variable array of cassettes (Fig 1A). The stable platform of the integron contains i) the integrase gene (*intI*) under the control of its promoter P_int_, ii) the *attI* integration site and iii) the P_C_ promoter of cassettes. The variable part consists of an array of cassettes, each of which is usually composed of a promoter less gene associated with a recombination site called *attC*. Only the first cassettes (closest to the P_C_ promoter) can be expressed, while the rest represent a low-cost reservoir of valuable functions for the cell. Many stresses, including the action of certain antibiotics, can trigger the integrase expression [5,6]. The integrase then catalyzes excision and integration of cassettes in the first position in the array, that is at the *attI* site, where they become expressed. The configuration of cassettes that enables the cell to escape stress will be selected. In this way, cassette shuffling enables the bacterium to rapidly screen for all the functions that optimize its survival in a given environment [4,7].

In the context of the ever-increasing pressure imposed by the overuse of antibiotics, integrons are regularly found to encode resistance genes to multiple antibiotics and associated with mobile genetics elements (conjugative plasmids and/or transposons) [8,9]. These integrons are called Mobile Integrons (MIs) and typically carry small arrays of cassettes probably facilitating their horizontal transfer. However, other integrons are sedentary and located on bacterial chromosomes (sedentary chromosomal integrons, SCIs) [10]. SCIs might be the ancestor from which the MIs evolved [11]. They can easily store dozens of cassettes (up to 301 in *Vibrio vulnificus*), encompassing a substantial fraction of their host genomes (up to 3%) [1,12]. The massive SCI from the seventh pandemic *V*. *cholerae* constitutes the paradigm in the field [10]. Among its 179 cassettes, several types of cassettes were found, such as cassettes with embedded insertion sequences like IS*3* (two cassettes) and IS*911* (one cassette). As with all the SCIs, most of the functions of the *V*. *cholerae* SCI remain unknown, except for the *catB9* cassette that confers chloramphenicol resistance and the 19 toxin–antitoxin (TA) cassettes [4,13–16]. As classical TA systems, those found in integrons carry their own promoters and encode a stable toxin targeting an essential cellular function and an antitoxin counteracting the action of the toxin [14,16,17].

Yet, except the TA cassettes, only the first few cassettes of SCIs are expressed, leaving more than 90% of the cassette array silent. This leads to question: how can such a massive structure that is plastic and mostly not expressed be stable within bacterial genomes over long evolutionary timescales?

In recent studies, we found that the orientation of SCIs towards replication was a crucial factor of their stability [18]. The reason for this lies in the form of the recombination substrates of the integron integrase. While this recombinase recognizes the *attI* site in its double-stranded (ds) form through its primary sequence, the *attC* sites are recombined in a single-stranded form [19–21]. Although both the bottom and top strands of an *attC* site can form a secondary structure, only the bottom strand of the *attC* site (*attC*_*bs*_), in its folded form, was found to be recombined by the integrase. This selectivity of the integrase for the bottom strand is essential for the correct orientation of the cassette upon integration at the *attI* site, allowing its expression by the P_C_ promoter [21,22]. It was shown that SCIs are preferentially oriented so that recombinogenic *attC*_*bs*_ are located on the leading strand template where replication is continuous, further decreasing the chance of these *attC*_*bs*_ being structured and recombined [23,24] (Fig 1B, top panel). By inverting the SCI of *Vibrio cholerae*, and thus locating the *attC*_*bs*_ on the lagging strand template, we showed that the rate of cassette excision increased dramatically due to a better structuration of the recombinogenic *attC*_*bs*_. This increased cassette excision activity compromises the integrity of the overall structure and also affects the growth of *V*. *cholerae* in a negative way, mainly due to the increased excision of TA cassettes [18]. Here, we assess how this increased integron plasticity due to high cassette excision rate can shape the evolution of SCIs. For that, we use the exceptional plasticity of the inverted SCI of *V*. *cholerae* as a model to examine the trade-off between evolvability and stability of SCIs. Through an evolution experiment, we demonstrate that the inverted SCI carrying-strain (SCI Inv strain) quickly restores its fitness defect and loses its high plasticity in two distinct ways. First, we find that most of the time inactivation of the integrase is rapidly selected, notably through a frameshift induced by an indel occurring in a DNA polymerase slippage hotspot within its coding sequence (CDS). Second, and more strikingly, we find that the array of the inverted SCI can in part recover its original more stable orientation, by homologous recombination between two insertion sequences (ISs) present in the array. In the first way, since integrase functionality is rapidly lost, the integrity of the cassette array would be barely affected. Nevertheless, it may be that these cassettes can still be excised and recruited by other MI integrase platforms carried, for example, by incoming plasmids [25]. In this case, in the long-term, the SCI cassette content could be irretrievably lost. In the second way, the SCI recovers its stability with no impact on its functionality, uncovering an elegant link between large genome rearrangements and evolvability of *V*. *cholerae*.

## Results

### Experimental evolution reveals rapid loss of the burden of a highly recombinogenic chromosomal integron

**Fig 2:**
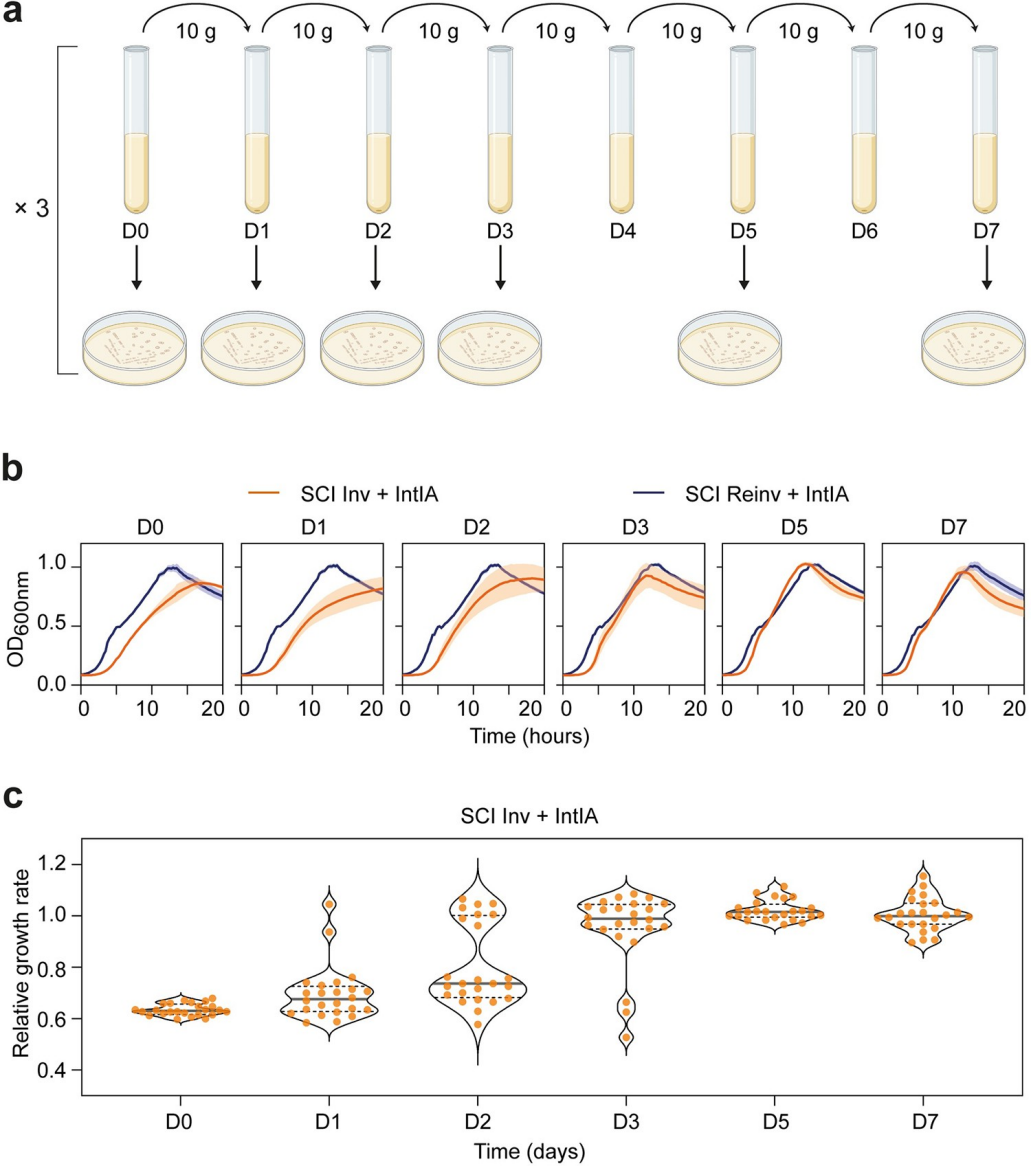
Experimental evolution of the SCI Inverted and Reinverted strains expressing integrase. **a.** Schematic overview of the evolutionary experiment. D0, D1, … stands for Day 0, Day 1, and so forth. After each day of growth (corresponding to 10 generations: 10 g), cultures were plated. 24 and 8 clones were collected for respectively SCI Inv and Reinv strains both expressing integrase (N = 24 and N = 8). Created with BioRender.com. **b.** Growth curves of the SCI Inv (orange lines) and SCI Reinv (blue lines) collected clones expressing integrase during the evolution experiment. For each curve, the line corresponds to the mean of the growth of the collected clones, and the shade corresponds to the standard errors at each timepoint. **c.** Distribution of the relative growth rates of the 24 collected clones at each time point of the evolution experiment. The growth rates of each SCI Inv clone for each day are represented as relative values compared to the mean growth rate of the SCI Reinv on the corresponding days. A violin plot is also represented to help visualize the bimodal distributions of the intermediate time points, as well as the median (full line) and quartiles (dotted lines) of those distributions.

To unmask the evolutionary constraints on chromosomal integrons and gain a better understanding of the trade-off between the evolvability and stability of SCIs, we carried out an evolution experiment using the exceptional plasticity of the inverted SCI of *V*. *cholerae*. Indeed, in our previous study, we demonstrated that inversion of the *V*. *cholerae* SCI led to a dramatic increase in its plasticity accompanied by a growth defect when the integrase is expressed [18]. Building on this, we postulated that the inverted SCI is an ideal model to explore the impact of increased cassette dynamics on its evolutionary trajectory. The SCI Inv strain containing a low-copy number plasmid expressing the integrase (pSC101::*intIA*) was used to initiate cultures in a medium that enabled the expression of the integrase (Fig 2A). Cultures were propagated daily for 7 days [26]. At the end of each day, D0, D1, D2, D3, D5 and D7 (respectively 0, 10, 20, 30, 50 and 70 generations), cultures were plated. 24 clones were collected, and the growth of each collected clone was measured (Fig 2B and S1 Table). As control, we also performed the same experiment using a strain in which the inverted SCI has been re-inverted to regain its original orientation (SCI Reinv strain) [18]. Note that for this SCI Reinv control strain, we only collected 8 clones for each day of evolution.

At D0, we observed a significant growth defect in the SCI Inv strain when the integrase is expressed compared to the corresponding SCI Reinv strain (Fig 2B and S1 Table). This severe growth defect is in line with what we observed in our previous study [18]. At each additional day of evolution, we observed a progressive attenuation of the SCI Inv growth defect. At D7, this defect disappeared completely, and the growth of both SCI Inv and Reinv strains no longer showed any significant difference. We determined the growth rate of each 24 collected clones for the SCI Inv strain (Fig 2C and S1 Table). At D0, all tested clones show a strong growth defect when expressing the integrase. The magnitude of the growth defect, as measured on the impact on the growth rate, is rather severe, with a decrease of the growth rate of about 35%. After 70 generations (D7), this growth defect disappears completely in every clone of each independent population (Fig 2C and S1 Table). We observed that some clones very rapidly lose the growth defect, recovering normal growth after only 10 generations (D1). After 30 generations (D3), only three of the 24 tested clones retained their growth defect, and 100% of the clones had lost the growth defect after 50 generations (D5). This evolution experiment shows that the cost associated with the inversion of the SCI in presence of the integrase is so high that it is strongly counter selected, leading to an amelioration after only a few dozen of generations.

### The integron integrase is rapidly inactivated in a highly recombinogenic chromosomal integron

**Fig 3:**
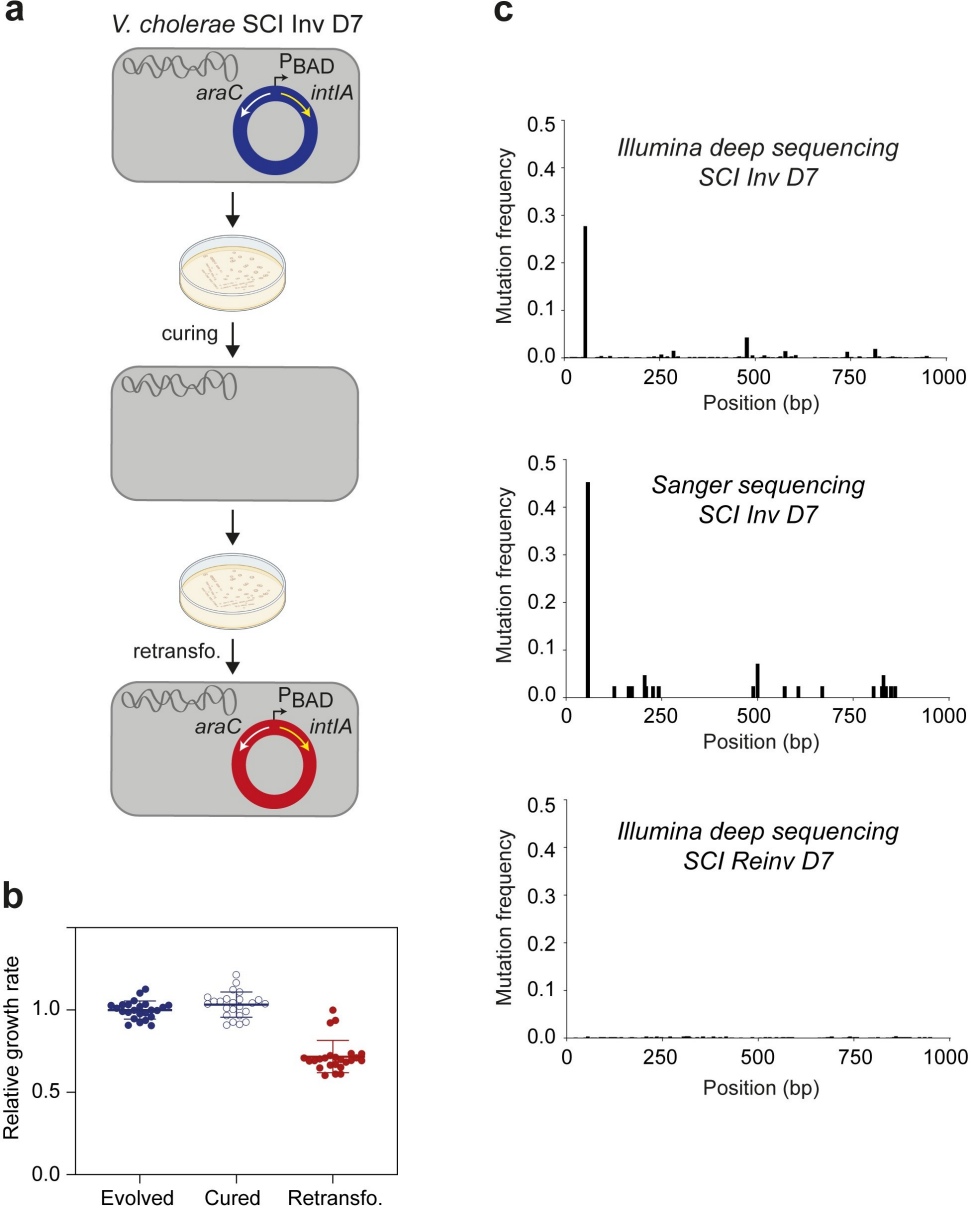
Analysis of the mutation pattern in the *Vibrio cholerae* integron integrase gene. **a.** Schematic overview of the plasmid curing and retransformation (retransfo.) The inducible P_BAD_ promoter, the *araC* repressor and the *intIA* integrase genes are respectively represented by black, white and yellow arrows. Created with BioRender.com. **b.** Distribution of the growth rates of the 24 clones before plasmid curing (Evolved), after plasmid curing (Cured) and after retransformation (Retransfo.) of the pSC101::*intIA*. The growth rates are represented as relative values compared to the mean growth rate of the 24 SCI Inv clones evolved from D7. **c.** Pattern of mutation of the *intIA* gene carried by the pSC101 vector. The x-axis is the nucleotide position relative to the adenine of the ATG start codon and the y-axis represents the mutation frequency. Each bar represents every type of variants (SNV: Single nucleotide variant; MNV: Multi-nucleotide *variant*; indel: insertion-deletion). The top panel shows results obtained with Illumina deep sequencing approach using the SCI Inv evolved D7 strain (with integrase). The middle panel shows results obtained with Sanger sequencing approach using the SCI Inv evolved D7 strain (with integrase). The bottom panel shows results obtained with Illumina deep sequencing approach using the SCI Reinv evolved D7 strain (with integrase). bp: base pair.

The growth defect of the SCI Inv strain was shown to be associated with high excision activity especially of TA cassettes [18]. Thus, the quick loss of growth defect was suggestive of a loss of the shuffling activity. To understand the suppression of the growth defect, we first sought to discriminate if the suppressor mutation occurred in the plasmid containing the integrase or if they were of another nature. To achieve this, we cured the plasmid of the 24 clones that were evolved for 70 generations (D7) (Material and methods and Fig 3A). The growth of the 24 SCI Inv clones, before and after the curing of the plasmid containing the integrase, was measured in a medium that enabled the expression of the integrase. The growth of those clones was not found to be different (Fig 3B and S2 Table). Upon retransformation with the plasmid carrying the *intIA* integrase gene, the growth defect was recovered in 21 clones out of 24 (Fig 3B and S2 Table). This showed that the suppressors of the growth defect arose mostly in the plasmid (21/24) but could also be associated with a modification of the chromosome (the remaining three clones). We first focused on the more common suppressors, i.e. located on the plasmid. To do that, we extracted the plasmids from the three bulk populations evolved for 70 generations, pooled them in equal proportions and sequenced this heterogeneous mix of plasmids to a high depth using an Illumina platform. We found that half of the mutations in the plasmid affected the regulation of the expression of the integrase, mostly through the inactivation of the *araC* gene that is essential to ensure gene expression through the P_BAD_ promoter (see the plasmid scheme Fig 3A, top panel). The rest was found to be in the *intIA* gene (Fig 3C, top panel and S2 Table). Many mutations were present at very low frequencies all along the sequence of the integrase, mostly indels or nonsense mutations. No substitution was found particularly enriched as it could have been expected in the catalytic domains of the integrase like modifications of active-site residues or of the catalytic tyrosine [27]. Instead, indels occurring in DNA homopolymers were found predominant. These indels produce a frameshift thus demolishing the entire integrase protein. One, a stretch of eight “A” (8-homopolymer) starting 57 bp after the ATG, was found to be mutated at a frequency of ~30% (Fig 3C, top panel and S2 Table). To confirm that this pattern of mutations found in the integrase did not come from a bias in the Illumina sequencing run, we also sequenced the integrase from 42 clones coming from the same three populations using classical Sanger sequencing (Fig 3C, middle panel and S2 Table). Even though it offers much less coverage, this approach led to the same general pattern of mutation with an over-representation of indels in the 8-homopolymer of “A” at the beginning of *intIA*. As control, we also extracted the plasmids from the three bulk populations evolved for 70 generations from the SCI Reinv strain. As expected, we observed very few mutations in the integrase gene (Fig 3C, bottom panel and S2 Table). Thus, we show that integrase mutation is the easiest way to recover from a growth defect associated with overactive recombination, and that this inactivation occurs primarily in a homopolymer motif.

**Fig 4:**
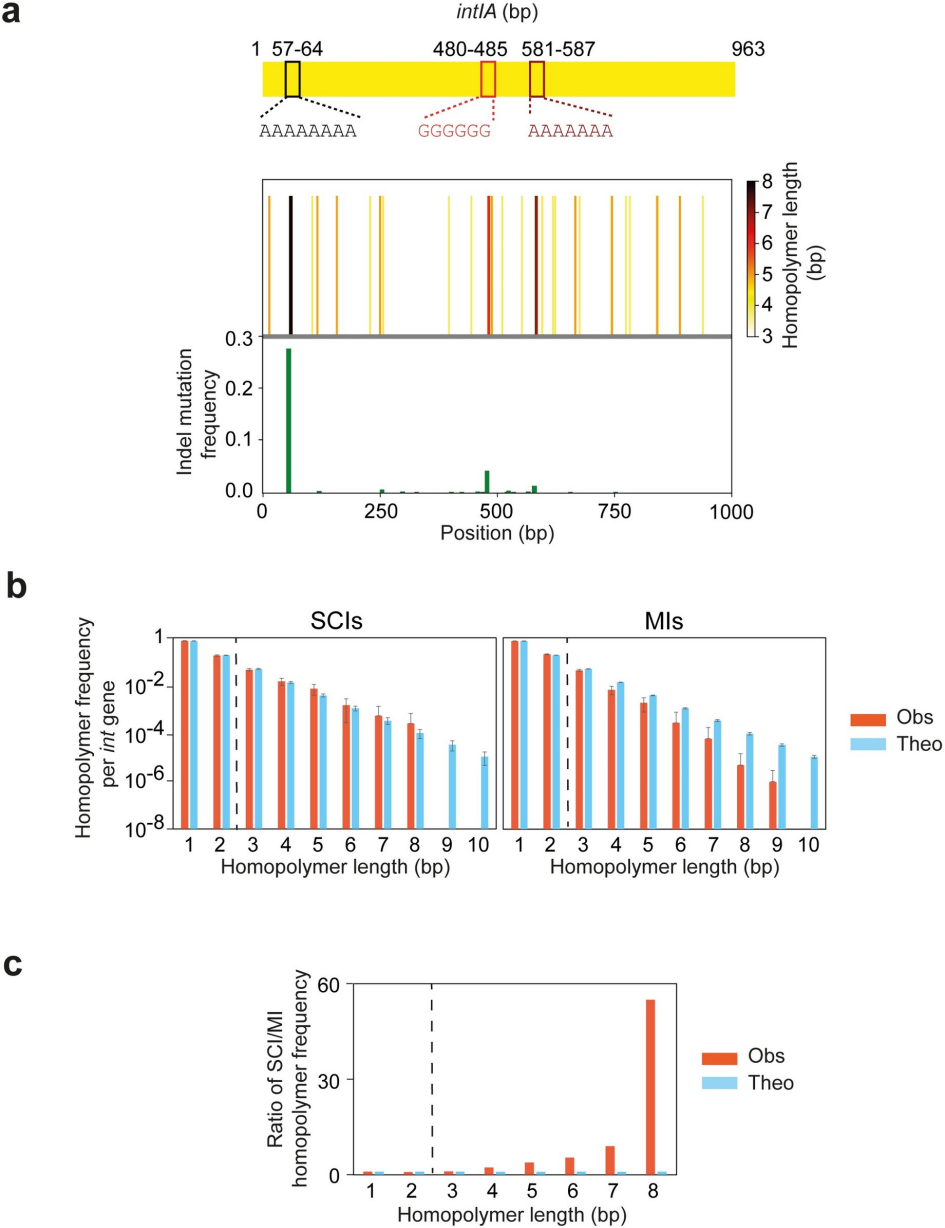
Analysis of the DNA homopolymer distribution among the integrase genes. **a.** Insertion-deletion (indel) mutation frequency and homopolymer distribution in the *intIA* integrase gene. A schematic view of the integrase gene and the position of the three largest homopolymers is shown just above the graph to help locate the mutation pattern. The top panel of the graph shows a barcode representing the distribution of homopolymers all along the *intIA* integrase gene with colors ranging from white to black depending on the length of the homopolymers, respectively from the smallest to the largest (from 3 to 8 bp). The bottom panel of the graph corresponds to the top panel of the Fig 3C in which mutations that do not correspond to indels in homopolymers have been removed. Note that we only considered the homopolymers containing three or more repeats. **b.** Frequency of the DNA homopolymers for lengths ranging from 1 to 10 within the integrase genes of SCIs (left panel) and MIs (right panel). For each panel, the frequency of the homopolymer lengths observed (obs) in SCI and MI integrase genes is represented in orange-red and the frequency of the homopolymer lenghts theoretically (theo) expected from randomized sequences is represented in light blue. The randomized sequences were generated by randomly shuffling the nucleotides of each SCI and MI integrase sequences. **c.** Ratio of the DNA homopolymer frequency for lengths ranging from 1 to 10 within the integrase genes of SCIs compared to the MI ones. The ratio observed (obs) between SCI and MI integrase genes is represented in orange-red and the ratio theoretically (theo) expected from the randomized sequences is represented in light blue. bp: base pair.

We precisely analyzed the distribution of homopolymers all along the *intIA* integrase gene and represented them in a “barcode” manner with the large homopolymers corresponding to the black color (Fig 4A and S3 Table). We attempted to correlate the indel frequencies found in the integrase gene with homopolymer distribution (Fig 4A and S3 Table). We found that the 8-homopolymer represents a hotspot of mutations whereas the two smaller 6 and 7-homopolymers attract mutations but at a much lower level.

We then wondered whether the presence of long homopolymers could be a characteristic of integron integrase genes. For that, we extracted the integrase sequences from the complete integrons identified with IntegronFinder 2.0 in a previous study (12). We searched for all the k-mer of nucleotides with k ranging from 1 to 10, termed homopolymers in the following for the sake of simplicity. We then plotted the frequency of the homopolymers in function of their length for SCI and MI integrase sequences (Fig 4B, right and left panels respectively and S3 Table) and compared each with the expected frequencies obtained from randomized sequences of the same SCI and MI integrase sequences. The distribution of the homopolymer lengths in SCI integrases is almost identical to the randomly simulated distribution. This means that long homopolymers have not been selected to exist in SCI integrases, but still their existence is important because they allow some slippages after SCI inversion. In contrast, the homopolymers in MI integrases are much shorter. A comparison of the homopolymer lengths of SCI versus MI integrases reveals that the 8-nucleotide homopolymers are 55 times less represented in MI integrases than SCI ones, whereas this differential is not observed in the corresponding randomized sequences (Fig 4C and S3 Table). This could reduce the probability of the MI integrases being inactivated by this way.

### Homologous recombination between insertion sequences helps to maintain chromosomal integron cassette arrays

**Fig 5:**
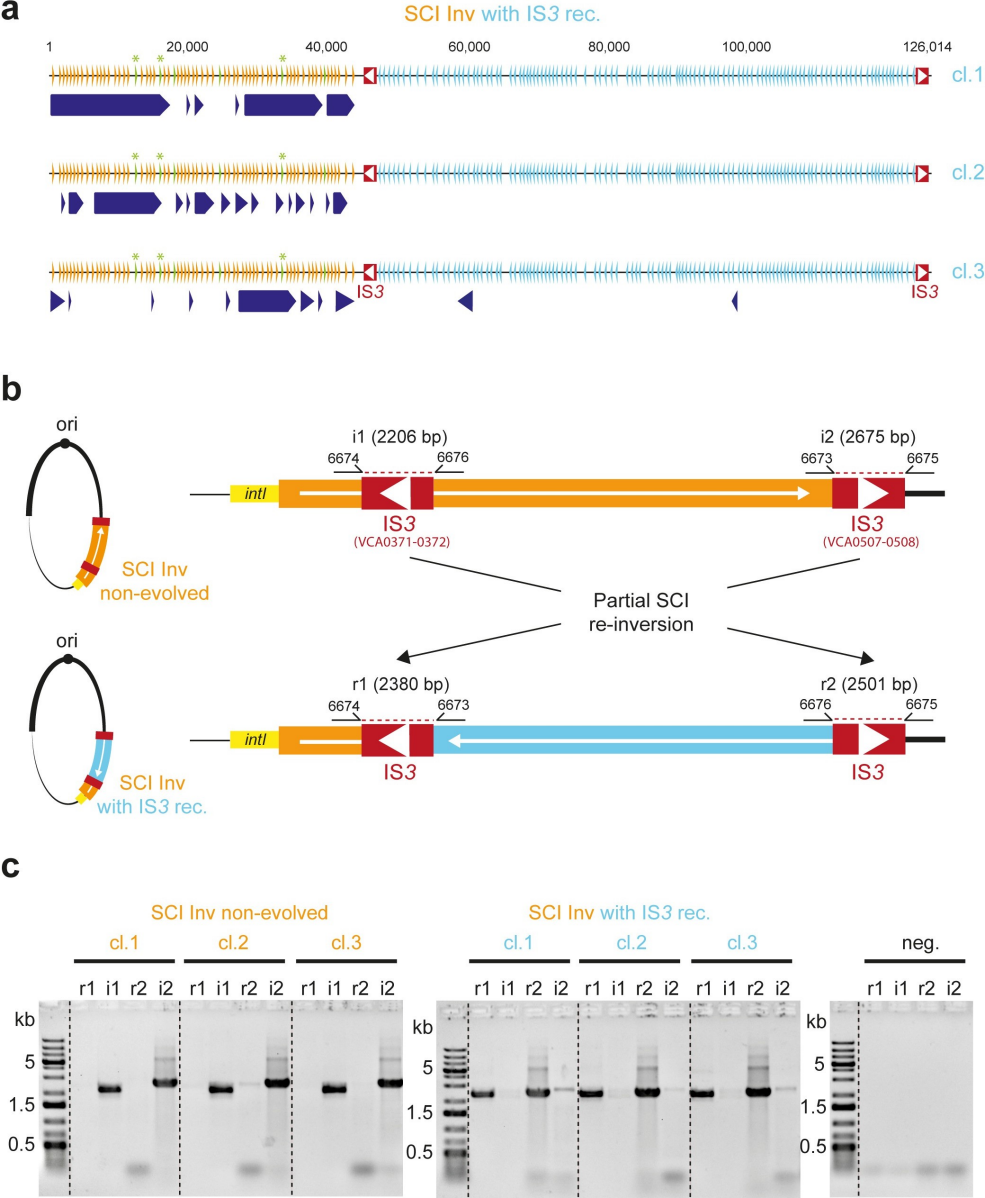
Analysis of the partial re-inversion event in the array of the *Vibrio cholerae* chromosomal sedentary integron. **a.** Representation of the SCI array of the three clones of interest. Orange triangles show the cassettes from the inverted part of the array, and the blue ones, the cassettes from the re-inverted part of the array (i.e. with IS*3* recombination, IS*3* rec.). The green triangles show the six toxin-antitoxin cassettes present in the first inverted part of the array. The duplicated ones are indicated by an asterisk. The dark blue arrows represent the deletion events. The red rectangles each with a white arrow show the IS*3* copies. **b.** Schematic representation of the partial SCI re-inversion event at the scale of the chromosome 2 of *V*. *cholerae* and focus on the two recombination points involved in this re-inversion. The PCR primer pairs used to identify the recombination event are represented: i1 and i2 are the primer pairs that should be positive in non-evolved. r1 and r2 are the primer pairs that should be positive upon recombination between the two IS*3* copies within the SCI (SCI inv with IS*3* rec.). **c.** PCR profile of the six clones tested for the SCI array configuration. The gels show the PCR results obtained using r1, i1, r2, i2 primer pairs for three non-evolved SCI Inv clones (left panel) as well as for each of the three evolved clones with IS*3* recombination (right panel).

Upon retransformation of the evolved clones with the *intIA* plasmid, we identified three SCI Inv clones, issued from two different populations, that retained an unaffected growth rate while the others recovered the characteristic growth defect of the SCI Inv strain (Fig 3A). This indicates that the suppressor mutations did not occur in the plasmid and *a fortiori* in the *intIA* integrase gene, but rather in the chromosome of these three clones. We therefore extracted their genomic DNA and queried the presence of mutations by whole genome sequencing. Illumina sequencing and subsequent variant calling using the sequana pipeline [28] showed the absence of any SNPs or indels in these clones, with the exception of SCI in which cassette deletions were found, as we would expect in SCI Inv strains where IntIA was expressed. We therefore asked whether the lack of recovery of growth defects in the three clones could be explained by their cassette content. The use of short reads does not allow an effective assembly of the SCI due to the numerous repeated sequences in that region, so we relied on the coverage of the different cassettes to assess their presence or absence (Fig 5A). The extensive deletion events that occurred in all three clones did not allow the identification of a specific cassette that was consistently excised in these clones and that could easily explain their phenotype. However, the cassette excision events observed in all three clones shared the same pattern: a high number of excisions in the first part of the array and almost no excisions in the second part of the array (Fig 5A). Interestingly, the boundary between these high and non-excision parts of the array was identical in all three clones. This boundary involved an insertion sequence (IS) from the IS*3* family that had previously transposed in the CDS of a cassette in the array (Fig 5A). This IS*3* contains two partly overlapping ORFs, as it is characteristic for the IS*3* family [29,30], with a total length of 1261 bp. Interestingly, a second copy of this IS*3* is also present in the SCI, inserted in the very last cassette of the array (Fig 5A). The two ISs are inserted in opposite divergent directions. In this configuration, recombination between the two copies of IS*3* would lead to the inversion of the fraction of the cassette array that separates them. We therefore reasoned that in the three clones in which no growth defect could be observed in the presence of the functional IntIA integrase, this event occurred so that most of the array of the SCI Inv was now re-inverted, which could explain the phenotype. We searched for this event performing PCR using a set of four primer pairs surrounding the two recombination points: one set for each recombination point and for each orientation of the cassette subset between the two copies of IS*3* (Fig 5B). We observed that in our three clones of interest, the PCRs which should be positive in the case of recombination between the two IS*3* copies (r1 and r2) were in fact positive and made up the major band (r1>i1 and r2>i2) (Fig 5C). In the three non-evolved clones, used as controls (Materials and methods), the PCRs that should be positive in the native inverted configuration were in fact positive and made up the major band (i1>r1 and i2>r2) (Fig 5C). In both evolved and non-evolved SCI Inv strains, we found a very weak amplification signal respectively for the i1 and i2 PCRs and the r1 and r2 ones while no amplification could be observed in the negative controls without DNA template. This means that the recombination event between the two IS*3*s, leading to the inversion of a 79,449 bp segment of the SCI array, probably occurs at a very low frequency whenever *V*. *cholerae* is cultivated, even in the absence of integrase, and is selected under conditions where it provides an advantage.

Since the recombination involves two IS*3* copies, we legitimately asked whether the recombination involved the seemingly functional transposases encoded by each copy. First, no target site duplication could be found on each side of the IRL and IRR of both IS*3* copies annotated as such by IS finder [30], meaning that no recent transposition event involving either copy of the IS*3* occurred. Second, we sequenced each of the previous PCR products and again found no target site duplication in the clones in which the inversion just occurred, indicating that the inversion involved homologous recombination and not transposition events. Interestingly, of the six TA cassettes present in the highly recombinogenic SCI inverted part in the three sequenced clones, we observed that the three non-duplicated TA3, TA4 and TA6 cassettes (in green in Fig 5A) were never excised in any of the three clones. In contrast, the other three duplicated TA1, TA2 and TA5 cassettes (in green with asterisk in Fig 5A) were found to be excised much more often. This observation fits with the previously described role of TAs, which acts through a “cassette loss killing” process that cannot be effective if TA cassettes are duplicated [18].

## Discussion

The maintenance of a large number of functions in SCIs represents an obvious long-term evolutionary advantage, given that each function could be expressed in response to critical stresses. Nevertheless, the precise mechanism underlying the second-order selection of these vast silent cassette arrays remains unknown. Our previous results had enabled us to sketch out quite precisely how SCIs maintain their large reservoir of cassettes in bacterial genomes [18]. This regulation occurs thanks to the presence, all along the SCIs, of a set of “special” cassettes–the TA cassettes–which become toxic when excised [18]. Therefore, all strains with highly recombinogenic SCIs are expected to exhibit a growth defect. This is what we previously described for our SCI Inv strain, in which the SCI has become very active thanks to the high folding capacity of all the recombinogenic *attC* strands, including those of the TA cassettes. In the present study, we addressed the question of how a cell could recover from such a dramatic phenotypic defect? Our results show that there are two viable options, one fairly obvious, namely inactivation of *intIA*, and the other, which we had not anticipated, involving genome rearrangements leading to a return to non-recombinogenic orientation of a large part of the cassette array.

In our previous results, we had observed that the growth defect of the SCI Inv strain was dependent on the integron integrase activity. Indeed, we had shown that the SCI Inv strain does not have any growth defect in absence of IntIA and that a catalytically active integrase was necessary to induce a growth impairment in that strain [18]. It seems therefore consistent that events affecting the *intIA* gene, prohibiting its expression or directly affecting its gene, are those most frequently selected to attenuate the growth defect. Concerning the integrase expression, the mutations found in the *araC* regulator of the used P_BAD_ promoter could be considered as an artefact of the experiment. Nevertheless, this confirms that silencing the recombination process abolishes the cost. If, in some genomes, integrase expression is controlled by certain regulators, this could be somewhat representative of this situation. Concerning the mutations affecting the integrase CDS, the most common are nonsense and indels, both of which lead to inactive truncated integrases. Interestingly, half of these mutations have been found in the 5’ part of the integrase gene located in an 8-homopolymer. This type of sequences, defined as short sequence repeat, has already been identified in prokaryotes as hotspots for slipped-strand mispairing (SSM) events during replication [31]. SSM is one of the mechanism responsible for phase variation, an adaptive process that accelerates the rate of spontaneous mutations in bacterial subpopulations, facilitating reversible phenotypic changes and rapid adaptation [32,33]. In *V*. *cholerae*, the phase variation modulating the expression of the O1 antigen enables this pathogen to generate various subpopulations of cells needed for escaping predation by O1-specific phage [34]. Here, we provide the first evidence of loss-of-function of one SCI integrase due to SSM events in response to the toxicity of unsustainable cassette dynamics that threatens the maintenance of large cassette reservoir arrays. This shows that loss-of-function is the main strategy for bacterial adaptation and that the supply of mutations is the key. We did not see any mutations in the integrase active site, but this is not surprising as there are many ways of inactivating a function without affecting the active site. In addition, the sequences corresponding to the active sites in a protein are generally robust, with only a couple of essential amino acids.

We also demonstrated, by simulating random sequences, that the homopolymers in the SCI integrase genes are not longer than expected, suggesting that there is no selection for their presence. This result is not surprising, since integrase expression, when SCIs are in their natural orientation, does not affect cell growth [18]. Interestingly, homopolymers in MI integrases appear to be shorter than theoretically expected. This could be explained by the fact that, contrary to SCI integrases, MI integrases are disseminated by the conjugation process. Indeed, the generation of single-stranded DNA during this DNA transfer process could make these homopolymers more susceptible to mutagenesis. This would lead to a counter-selection of these homopolymers favoring integrase inactivation in the MI sequences. These observations align with mathematical models suggesting that functional MI integrases can endure within a population despite significant fitness costs [35]. This resilience stems from MIs requiring optimal integrase activity to ensure high degree of combinatorial phenotypic diversity thus enabling the population to respond rapidly to shifting selective pressures. This evolutionary set up would be one of the keys to the success of MIs.

The second observed outcome of the evolution experiment–the spontaneous re-inversion of a substantial part (63%) of the SCI cassette array by homologous recombination between two IS*3* copies–was less expected. We previously showed that the inversion of the SCI led to a higher excision rate of the cassettes by placing the *attC*_*bs*_ on the lagging strand template. Hence, re-inversion of the SCI brings the *attC*_*bs*_ back to their native and less recombinogenic state (i.e. on the leading strand template). The portion of the observed re-inverted array contains ten non-duplicated TA systems versus only three in the other part [16]. Indeed, the increased probability of excision of a TA cassette leading to cell death or cell cycle arrest, exerts a strong selective pressure for the re-inversion of the section of the array that concentrates the most TA cassettes. Furthermore, the partial re-inversion of the SCI array is another possible means of suppressing the growth defect in the SCI Inv strain expressing the integrase, since it is not the integrase that is intrinsically toxic but the increased excision of the TA cassette. This evolutionary solution has the advantage of keeping integrase functionality intact. Note that we have already observed this type of recombination between repeated IS elements, leading to the fusion of the two chromosomes of *V*. *cholerae* [36]. However, this is the first time that we observed such a large chromosomal rearrangement inside the *V*. *cholerae* SCI structure, demonstrating an important role of the duplicated ISs present along the SCIs. Using a bioinformatics prediction, we determined the location of IS*3* in the SCIs of 83 *V*. *cholerae* genomes. The pattern of IS*3* distribution found earlier in our *V*. *cholerae* N16961 strain (i.e. IS*3* located in the 5’ part of the SCI and a second copy at the end, Fig 5) is not a generality among the *V*. *cholerae* species. However, this peculiar pattern is conserved in *V*. *cholerae* strains from the 7^th^ pandemic (which are highly clonal), as well as in pre-pandemic *V*. *cholerae* strains (S1 Fig).

Interestingly, whatever the two distinct ways of escaping–“Belt and Braces”–we demonstrated that the inverted SCI-carrying strain quickly select a solution to restore its fitness (less than 70 generations).

These results illustrate a tight control of SCI functioning, favouring cassette stability and maintenance over evolvability, orchestrated by a fascinating interplay between gene orientation, single-stranded DNA, bacterial replication, toxin-antitoxin systems, site-specific recombination, loss-of-function mechanisms, genome rearrangements and bacterial fitness.

## Materials and methods

### Bacterial strains, plasmids and primers

The different bacteria strains, plasmids and primers that were used in this study are described in Tables 1, 2, and 3.

**Table 1:** Bacterial strains used in this study.

Strain number	Relevant genotypes or description	References
**Bacterial strains**		
I992, I993, J341	SCI inverted. *attC*_*bs*_ on lagging strand template (SCI Inv strains)	[18]
J053, J054, K025	SCI re-inverted into its original orientation, *attC*_*bs*_ on leading strand template (SCI Reinv strains)	[18]
**Transformed strains**		
K483, K485, K487	SCI Inv + pJ504	[18]
K493, K495, K497	SCI Reinv + pJ504	[18]
**Evolved strains**		
ER332	K483 culture evolved during 70 generations	This study
ER334	K485 culture evolved during 70 generations	This study
ER336	K487 culture evolved during 70 generations	This study
ER338	K493 culture evolved during 70 generations	This study
ER340	K495 culture evolved during 70 generations	This study
ER342	K497 culture evolved during 70 generations	This study
ER229-236	8 clones isolated from ER332	This study
ER237-244	8 clones isolated from ER334	This study
ER245-252	8 clones isolated from ER336	This study
ER253-260	8 clones cured from ER229-236	This study
ER261-268	8 clones cured from ER237-244	This study
ER269-276	8 clones cured from ER245-252	This study
ER277-284	8 clones transformed from ER253-260	This study
ER285-292	8 clones transformed from ER261-268	This study
ER293-300	8 clones transformed from ER269-276	This study
ER179-ER187-ER286	3 clones SCI inv with IS*3* rec	This study

**Table 2:** Plasmids used in this study.

Plasmid number	Plasmid description	Relevant properties and construction
pJ502	pBAD43*ΔattC*_*aadA1*_	*oripSC101*; [Sp^R^] [18]
pJ504	pBAD43 *ΔattC*_*aadA1*_::*intIA*_*Vch*_	*oripSC101*; [Sp^R^] [18]

**Table 3:** Primers used in this study.

Primers	Sequences
1440	CTGCAGCCCGGGGGATCCAC
1441	GAATTCGATATCAAGCTTATCGATAC
6673	GAAGAACCACGAGTAGAAGAGTATGGG
6674	GGACTCCTATTATACCCGCCTGTC
6675	GCCTGTAGCAATGGCAACAACGTTG
6676	GCTTCACCAGTGTTCCCTACTTCTTG

### Media

*Vibrio cholerae* strains were grown in Luria Bertani (LB) at 37°C. Glucose (Glc), L-arabinose (Ara) and fructose (Fruc) were added respectively at final concentrations of 10, 2 and 10 mg/mL. The spectinomycin (Spec) was used at the following concentrations: 100 μg/mL in absence of glucose and 200 μg/mL in presence. To avoid catabolic repression during various tests using arabinose as inducer for the expression of the integrase, cells were grown in a synthetic rich medium: MOPS Rich supplemented with fructose (1%) as a carbon source.

### Evolution experiment

Three clones each stemming from different biological replicates of the SCI Inv and SCI Reinv strains containing the low-copy number pSC101 vector, carrying the *intIA* gene under a P_BAD_ promoter, were grown overnight (O/N) in LB + Spec + Glc. These first cultures correspond to that we called the Day 0 (D0) meaning 0 generation of evolution. To initiate the evolution experiment, each culture was diluted at 1:1000 in 5 ml of the inducing medium (MOPS + Spec + Fruc + Ara). Agitation was set to 180 rpm. Cultures were allowed to grow for 24 h at 37°C (resulting to 9.97 generations). This was repeated six times, that is with a total of ~70 generations (~10 generations every 24h). Moreover, after 0 (Day 0), 10 (Day 1), 20 (Day 2), 30 (Day 3), 50 (Day 5) and 70 (Day 7) generations, the six independent cultures were streaked on LB agar plates containing Spec + Glc. For the SCI Inv strains with integrase, we isolated 24 independent and randomly chosen clones (8 for each biological triplicates) and for the SCI Reinv strain with integrase, we isolated 8 clones (3, 3 and 2 for each biological triplicates). Each of these clones were grown O/N in LB + Spec + Glc and then used to perform automated growth rate measurements.

### Automated growth rate measurements

O/N cultures of the indicated strains (see above) were diluted 1/1000 in MOPS + Spec + Fruc + Ara medium and then distributed by quadruplicate in 96-well microplates avoiding the use of external rows and columns. Automated growth-curve were generated using a TECAN Infinite microplate reader, with an absorbance measurement of 600 nm taken at 10-min intervals at 37°C on maximum agitation for 15 h. Maximum growth rates during exponential phase were directly obtained using the “GrowthRates” R package. The package can be downloaded from CRAN [https://cran.r-project.org/package=growthrates].

### Plasmid curing

In the evolution experiment, the integrase was carried on a pSC101 plasmid and under the control of a P_BAD_ promoter. To replace this plasmid by a new one, we took advantage of the natural instability of plasmids in *V*. *cholerae* [37]. 24 isolated clones from the three biological replicates evolved after 70 generations were cultivated O/N in LB + Glc (without spec) and then plated on LB agar + Glc plates. The resulting clones were streaked on LB agar + Glc and in parallel on LB agar + Spec + Glc plates. The clones growing on the first but not on the latter where the ones in which the plasmid was cured, which was confirmed by the absence of PCR amplification using the 1440 and 1441 primers. For each of the 24 cured clones obtained, a new plasmid was added by electroporation.

### Electroporation in *V*. *cholerae*

Cells were grown to late exponential phase (OD_600_ ~0.7), then 10 mL was pelleted. The pellet was washed twice in G buffer (137 mM Sucrose, 1mM HEPES, pH 8.0). 50 μL of the resulting cell suspension was mixed with ~250 ng of the pSC101::*intIA* (pJ504) plasmid in a 1 mm electroporation cuvette. Electroporation was performed at 2000 V. Electroporated cells were grown at 37°C in LB + Glc for 1 h for phenotypic expression before plating on LB agar + Spec + Glc plates. The presence of the plasmid was checked by PCR amplification using the o1440 and o1441 primers.

### Plasmid DNA extraction

At the end of the evolution experiment (70 generations), the *intIA*-carrying plasmids of the three independent populations were extracted using the Thermo Fisher Miniprep Kit. The three plasmid preparations were adjusted to 50 ng/μL as measured by Qubit, and then mixed at equal proportion prior to Illumina sequencing. The same Kit was used for plasmid extraction from the corresponding isolated clones prior to Sanger sequencing.

### Genomic DNA extraction

Clones that were to be sequenced or checked by PCR for genome rearrangements were cultivated overnight in LB + Spec + Glc. The genomic DNA was extracted following the specification of the Qiagen gDNA extraction Kit. DNA concentration was assessed by Qubit and quality assessed by migration on a 1% Agar gel prior to sequencing.

### PCR checking of partial SCI re-inversion

Partial SCI re-inversion was checked by PCR using primer pairs i1 (6674 and 6676), i2 (6673 and 6675), r1 (6674 and 6673) and r2 (6676 and 6675). For the partial SCI re-inverted clones, bands are expected with the r1 and r2 primer pairs (respectively 2380 bp and 2501 bp) and for the non-evolved SCI Inv clones (no partial SCI re-inversion), bands are expected with i1 and i2 primer pairs (2206 bp and 2675 bp respectively). Note that non-evolved SCI inv clones correspond to clones selected from the D0 cultures (obtained in a medium that does not enable integrase expression, i.e. in the presence of Glc).

### Sequencing and SNP calling

Both the plasmidic and genomic DNA were sequenced using an Illumina MiSeq sequencer with paired end 150 bp reads (2 × 150 bp). The obtained sequenced data were then analysed using the Sequana variant calling pipeline v1.1.2 [28], which is a Snakemake-based pipeline provided in Sequana v0.16.5. This pipeline involves an initial mapping step performed with the Burrows-Wheeler Alignment software (bwa v0.7.17) [38] using default parameters. Then, the variant calling was conducted using the Freebayes software v1.2.0 [39], which maps the sequencing data and identifies variants. A mutation frequency threshold of 0.1% was applied based on a median coverage of 10,600X along the plasmid’s sequence. Furthermore, the analysis incorporated an assessment of strand balance to validate the detected variants. A Fisher test was used to filter out variants whose strand balance deviated from 0.5, thus contributing to the robustness of the results. A Python notebook used to process the variant calling files is available at https://github.com/biomics-pasteur-fr/manuscript_belt_and_braces/.

### DNA homopolymer analysis in integron integrase genes

The integrase sequences were extracted from the complete integrons identified with IntegronFinder 2.0 [12,40]. We classified them as "mobile" and "sedentary" based on the length of the cassette array, as suggested by the authors. The Nextflow pipeline used to analyze the DNA homopolymers within the extracted integrase genes is available at https://gitlab.pasteur.fr/gmillot/homopolymer and is briefly described here. For each SCI and MI integrase sequence of the input batch: 1) the sequence is split according to homopolymers (e.g., ATTTAACC is split into A, TTT, AA, CC), 2) homopolymer lengths were enumerated, independently of nucleotide content, which returns the observed counting and related proportions of homopolymer lengths in the sequences, 3) the two first steps were re-applied after randomly shuffling the nucleotides of each sequence, 4) This was performed 10,000 times and means of homopolymer lengths were computed, which returns the theoretical counting and related proportions of homopolymer lengths in the sequence. All the homopolymer results are available at https://doi.org/10.5281/zenodo.8305871.

### IS*3* location pattern analysis in the SCI of multiple *Vibrio cholerae* genomes

#### Genome dataset

We analyzed whether the specific location pattern of IS*3* along the SCI of *V*. *cholerae* N16961 could be found in other *V*. *cholerae* genomes. As a starting point, we focused on the *V*. *cholerae* part of the SCI dataset previously generated in [18]. Briefly, this comprises 86 completely assembled *V*. *cholerae* genomes from the NCBI RefSeq database of reference microbial genomes (downloaded on March the 30^th^ of 2021). For each genome, integrons were predicted using IntegronFinder 2.0 and SCIs were defined as complete integrons or CALINs with at least 11 predicted *attC* sites.

#### IS3 prediction and SCI visualization

We predicted IS*3* in the replicons harboring an SCI, using ISEScan v1.7.2.3 [41]. We visualized the location of IS*3* in each SCI using a matplotlib v3.4.3 (https://ieeexplore.ieee.org/stamp/stamp.jsp?tp=&arnumber=4160265)-based homemade-script available at (https://gitlab.pasteur.fr/elittner/sci-is3-analysis). Briefly, we normalized the length of each SCI between its start and end positions. We also re-oriented all the SCIs such that the integrase is located on the left-hand side of the figure. ISEScan was unable to predict the orientation of some IS*3*, owing to an inaccurate calling of the transposase coding sequence. We reported such cases as “unknown orientation” in the figure.

#### Vibrio cholerae phylogeny

To order *V*. *cholerae* genomes, we built a core-genome phylogeny using the serial modules of PanACoTA v1.3.0 [42]. Briefly, we clustered all the genomes by computing MASH distances between all pairs and clustering the closely related ones by transitivity (MASH distance<0.0001) [43]. To remove redundancy, one genome per cluster was kept for further analysis. We removed from the analysis three divergent genomes, mis-classified as *V*. *cholerae* in RefSeq at the time they were downloaded (FORC_076, GCF_004117115.1; 4295STDY6534200, GCF_900324435.1; NCTC 30, GCF_900538065.1). We computed the species’ pangenome using MMseqs2 Release 13–45111 [44] and using a threshold of at least 80% amino acid identity and 80% coverage among genes belonging to the same pangenome family. Then, the species’ persistent genome was defined as the set of pangenome families having a unique member in at least 90% of the genomes. Using MAFFT [45], we aligned individually each pangenome family belonging to the persistent genome (alignments in proteins where each amino acid was replaced by the original codon). Finally, the resulting codon alignments were concatenated, and the phylogenetic tree was inferred with IQTree 2.2.2.2, using the option ModelFinder and computing 1000 ultrafast bootstraps. We included in the tree all the members of a same MASH-cluster with a zero-distance to the cluster representative.

## Supporting information

S1 Table Raw data corresponding to Fig 2. (XLSX)

S2 Table Raw data corresponding to Fig 3. (XLSX)

S3 Table Raw data corresponding to Fig 4. (XLSX)

S1 Fig IS*3* location pattern analysis in the SCI of multiple *Vibrio cholerae* genomes. Schematic overview of the SCIs belonging to complete *Vibrio cholerae* genomes available in NCBI RefSeq. Each row depicts the SCI of a single *Vibrio cholerae* strain. The lenght of each SCI is normalized between its start and end positions. In the few cases where the SCI has split into several parts, the interspace region is shown in white. The SCI lenght is then normalized between the startposition of its first part and the end position of its last part. Strains are ordered according to their position in a *Vibrio cholerae* core-genome phylogenetic tree, rooted with the outlier *Vibrio tarriae* RFB05. Highly clonal strains belonging to the 7^th^ pandemics are indicated in purple.
(EPS)
